# Cytokine delivery and targeting strategies in pet dogs with cancer: a comparative immuno-oncology approach

**DOI:** 10.3389/fimmu.2026.1906933

**Published:** 2026-07-06

**Authors:** Matheus Moreno Passos Barbosa, Timothy M. Fan

**Affiliations:** 1 Department of Pathobiology, University of Illinois Urbana-Champaign, Urbana, IL, United States; 2 Department of Veterinary Clinical Medicine, University of Illinois Urbana-Champaign, Urbana, IL, United States; 3 Cancer Center at Illinois, University of Illinois Urbana-Champaign, Urbana, IL, United States

**Keywords:** cancer immunotherapy, comparative oncology, cytokine therapy, intratumoral immunotherapy, targeted cytokine therapies, translational oncology

## Abstract

Cytokine therapies have long been hampered by a fundamental pharmacological problem: systemic absorption and distribution drives immune-related toxicity which precludes the achievement of therapeutic concentrations within tumors to mediate anti-tumor immunity. Engineering cytokines with tunable biodistribution properties to remain within or near the injection site has reinvigorated opportunities to explore cytokine-based immunotherapies for clinical evaluation. Pet dogs with spontaneous cancers offer a biologically rich and informative model system for investigating new therapeutic delivery strategies. Pet dogs develop tumors naturally in an immunocompetent setting, share key features of human tumor immunobiology and immune system architecture, and display similar pharmacodynamic immune biomarkers used for assessing tolerability and antitumor activities in human cancer patients. This mini review examines targeted cytokine delivery platforms evaluated in pet dogs with naturally occurring malignancies, including aluminum hydroxide-anchored IL-12, collagen-binding IL-2/IL-12 fusion proteins, antibody-cytokine immunocytokines (NHS-IL12 and hu14.18-IL2), PEGylated TNF-α, liposomal IL-2, plasmid IL-12 electrogene therapy, oncolytic viruses engineered to express IL-12 or IFN-β, and emerging delivery platforms. Solid malignancies studied in pet dogs include melanoma, soft tissue sarcoma, mast cell tumor, osteosarcoma, high-grade glioma, adenocarcinoma, fibrosarcoma, and urothelial carcinoma. Across diverse technological platforms, several pharmacodynamic patterns observed in dogs have also been recapitulated in first-in-human trials, emphasizing the potential wealth of a comparative oncology framework for evaluating novel immunomodulatory strategies. These parallels underscore the value of canine immuno-oncology trials in defining safety and biological activity for engineered cytokine constructs, with direct implications for guiding the development of dose selection and biomarker discovery in the human immuno-oncology field.

## 1 Introduction

Comparative oncology is the transdisciplinary study of naturally occurring tumors in multicellular organisms that model cancerous pathologies in human beings (1). While traditionally reliant upon rodent models, the value-added rationale for including pet dogs to inform human cancer investigations is based upon companion dogs sharing the same environment as human beings, having well-annotated and outbred genetics, and importantly developing tumors spontaneously in an immunocompetent setting (2, 3). While not universally applicable to all tumor histologies, comparative oncology studies have demonstrated that specific cancers including lymphoma, osteosarcoma, melanoma, urothelial carcinoma, breast carcinoma, glioma, and various soft tissue sarcomas, exhibit important biological and clinical similarities between dogs and humans (4–6). Additionally, standard of care for certain cancer types in dogs remains undefined, and permits the ethical evaluation of investigational therapies that are scientifically grounded and medically justified. Together, these opportunities afforded through a comparative oncology approach through inclusion of pet dogs, creates a powerful platform for translational research, allowing investigators to evaluate novel cancer therapeutics to inform human applications and also develop newer therapeutic options for veterinary species (3, 7, 8).

Comparative oncology investigations can be leveraged to address critical gaps in the drug development pipeline (9). Studies in tumor-bearing dogs allow researchers to examine critical aspects of drug development including PK/PD, mechanism of action, and dose-response relationships (9). Additionally, inclusion of pet dogs in anticancer discovery efforts allows for the humane collection of serial tumor biopsies, as well as the ability to recognize nuanced constitutional signs (i.e., fatigue or lethargy) associated with therapeutic interventions that might be undetectable in conventional, non-companion animal model systems. Complex immunobiologic insights gleaned from pet dog investigations might not be faithfully recapitulated in mouse tumor models due to limited tumor heterogeneity and differences in immune biology (10). In fact, close homology of the immune system components between human and dog affords a powerful opportunity to hypothesis-test immune modulatory strategies (3, 11), and moreover shared tumor heterogeneity, metastatic patterns, and immune landscape further bolster the relevance of evaluating immunotherapies in pet dogs, either contemporaneously with or as a prelude to future human clinical trials. However, several canine immune cell-populations remain less characterized than in humans, which can limit mechanistic interpretation.

Within this translational framework, cytokines represent a class of immunotherapeutic agents well suited to be evaluated through a comparative oncology approach. These pleiotropic immune signaling proteins are capable of activating lymphocytes, promoting tumor cell killing, and reshaping the tumor microenvironment (TME). Cytokines exert anticancer immune activities and have been clinically investigated for several decades. Despite years of developmental research, only three cytokine-based agents have received FDA approval for the treatment of specific human malignancies under narrowly-defined indications and include: interferon-alpha-2b (IFN-α2b), approved for hairy cell leukemia and melanoma (12, 13); high-dose interleukin-2 (aldesleukin), approved for metastatic renal cell carcinoma (1992) and metastatic melanoma (1998) (14, 15); and most recently, the IL-15 superagonist N-803 (nogapendekin alfa inbakicept), approved for BCG-unresponsive non-muscle-invasive bladder cancer (2024) (7, 8).

While these examples underscore the potential of cytokines for treating human cancer patients, the collective impact and clinical translation of cytokine therapies have been largely unrealized and limited by on-target, off-tumor systemic toxicity. For example, high-dose IL-2 is associated with life-threatening vascular leak syndrome, hypotension, and multiorgan dysfunction (16). More broadly, the rapid systemic distribution of unmodified cytokines leads to immune-related adverse events (e.g., cytokine release syndrome), which constrain the therapeutic doses achievable with the TME. These limitations have driven the development of engineered and targeted cytokine strategies designed to concentrate activity within the TME, thereby maximizing local immunostimulatory effects while minimizing systemic toxicity (17). Given the comparable anatomic size, metabolism, and immune composition of dogs with humans, pet dogs with naturally occurring tumors can serve as a biologically rich model for human cancers for evaluating next-generation, cytokine-based strategies, as will be discussed below.

**Figure 1:**
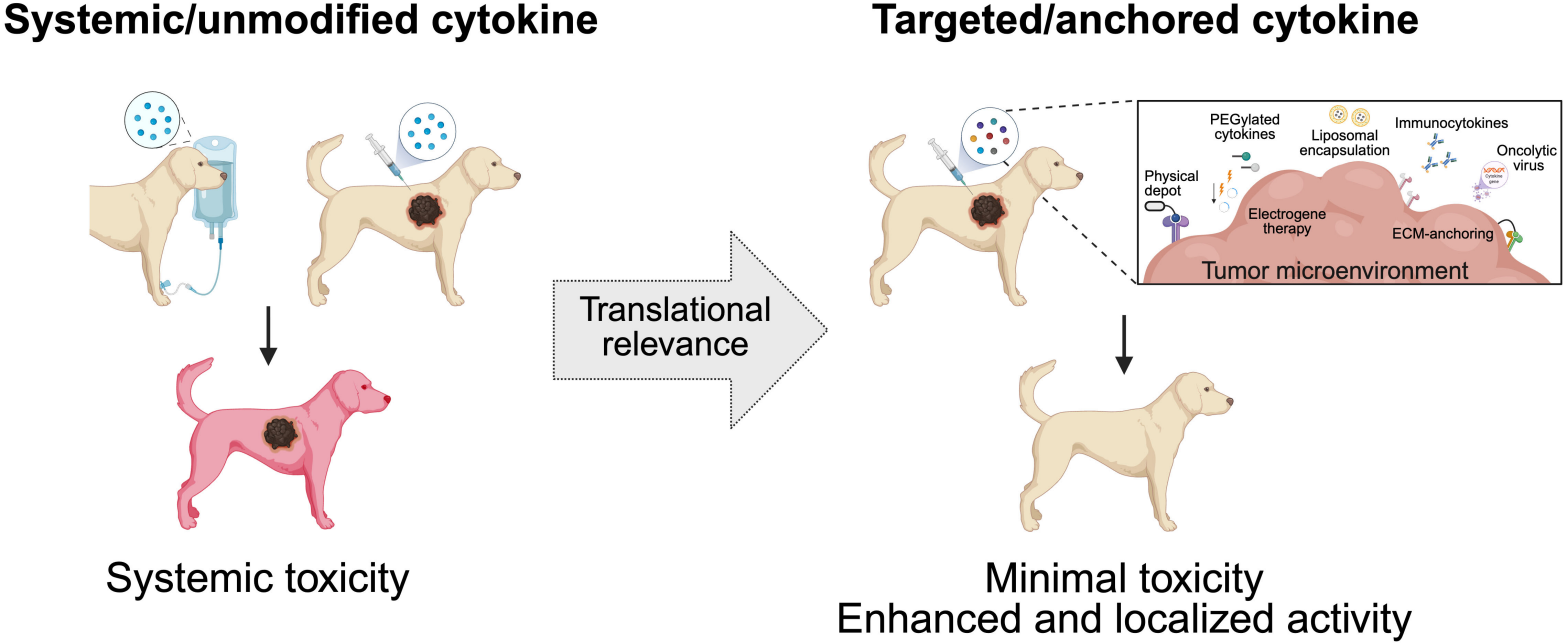
Targeted cytokine delivery strategies reduce systemic toxicity and enhance local antitumor activity. Left panel: systemic or unmodified cytokine administration, whether intravenous or intratumoral, results in widespread cytokine distribution and systemic toxicity. Right panel: targeted and anchored cytokine delivery strategies confine cytokine activity to the tumor microenvironment, resulting in minimal systemic toxicity and enhanced local immune activity. (Created with BioRender.com).

In veterinary oncology, cytokines have been investigated since the mids-1990s, with early research studies evaluating their activities against mast cell tumor, transmissible venereal tumors, malignant melanoma, and metastatic osteosarcoma (18–20). Clinical exploration of IL-2, IL-12, IL-15, and more recently engineered cytokine technologies has expanded with the recognition that unmodified cytokines share the same unfavorable side effect profile as in human oncology, being systemic toxicity driven by off-tumor immune activation restricts the safe clinical usage of untargeted cytokine therapies (21, 22). Modifying how and where cytokines are delivered and retained may help to maximize their antitumor activity without triggering the systemic pro-inflammatory responses that limit their clinical application (**Figure 1**). This mini review summarizes targeted cytokine strategies investigated in pet dogs with spontaneous tumors and focuses on innovations designed to improve intratumoral retention and anti-tumor activity, as well as highlights how these comparative canine studies have contributed to the broader translational pipeline for human cancer patients (**Table 1**).

**Table 1:** Summary of targeted cytokine delivery strategies evaluated in pet dogs with spontaneous cancer.

Strategy/agent	Species & clinical setting	Key veterinary findings	Translational impact and human clinical development
Aluminum hydroxide depot JEN-101 (ABP-IL-12)	Dog Melanoma	Safe; intratumoral CD3+ T cell infiltration and clinical responses without systemic AEs; <1% ABP detected transiently in circulation.	ANK-101 (tolododekin alfa); Phase 1, advanced solid tumors (NCT06171750)
Collagen anchoring LAIR1-IL-2/IL-12	Dog STS; melanoma	Median OS 256 vs. 65 days (historical) in melanoma; CD3+ T cell infiltration; effector gene expression re-engaged by second dose	CLN-617 (LAIR2-IL-2/IL-12); Phase 1 ± pembrolizumab (NCT06035744)
Electrogene therapy IL-12 plasmid	Dog MCT; melanoma; sarcoma	Nodule reduction within 1–2 weeks; increased lymphocytic infiltration; no mast cell degranulation or systemic AEs in MCT	Phase 1, melanoma (44); Phase 2 + anti-PD-1 (46)
Immunocytokine NHS-IL-12	Dog Melanoma	Serum IFN-γ >100 pg/mLpredicts toxicity; tolerable dose 0.8 mg/m²; 2 PR + 1 durable response (>9 months)	M9241; Phase 1 (NCT01417546); Phase 1b + avelumab (NCT02994953)
Immunocytokine hu14.18-IL2 (GD2-directed)	Dog Melanoma	1 CR + 1 PR at day 60 (n = 12); increased intratumoral lymphocytic infiltration	Phase 1/2, melanoma (61); Phase 2, neuroblastoma (62)
PEGylated cytokine PEG-hTNF-α	Dog Multiple tumor types	MTD 26.7 µg/kg; t½ ≈15 h; increased intratumoral necrosis and tumor blood flow; 1 PR (angiosarcoma)	Established extended half-life and antitumor necrotic effects vs. unmodified TNF-α in murine models
Liposomal encapsulation (inhaled and intravenous) IL-2 liposomes; liposome-DNA/IL-2 complexes	Dog Pulmonary metastases; lung carcinoma	2/4 osteosarcoma dogs: complete pulmonary regression (>12 and >20 months); increased BAL cytolytic activity (inhaled IL-2 liposomes); 3/20 dogs with lung metastases responded to IV liposome-DNA/IL-2, with improved OS vs. historical controls	Inhaled IL-2: Phase 1 sarcoma (66); 5/27 CR in melanoma (77)
Oncolytic virus M032 (oHSV-1-IL-12)	Dog High-grade glioma	Median OS 151 days vs. historical controls; no dose-limiting toxicities; intratumoral immune modulation mRNA signatures	Phase 1, glioblastoma (NCT02062827; NCT07076498)
Oncolytic virus VSV-cIFNβ-NIS	Dog Osteosarcoma	Higher intratumoral T cell immune signature associated with prolonged survival (VIGOR study)	Safety confirmed in healthy beagles (71) and client-owned dogs (72)

ABP, alum-binding peptide; AE, adverse event; BAL, bronchoalveolar lavage; CR, complete response; IFN-γ, interferon-gamma; LAIR, leukocyte-associated immunoglobulin-like receptor; MCT, mast cell tumor; MTD, maximum tolerated dose; NIS, sodium-iodide symporter; oHSV-1, oncolytic herpes simplex virus type 1; OS, overall survival; PEG, polyethylene glycol; PR, partial response; STS, soft-tissue sarcoma; t½, half-life; VSV, vesicular stomatitis virus.

## 2 Targeted cytokine delivery and engineering strategies in canine oncology

### 2.1 Aluminum hydroxide-based cytokine depots

Anchoring cytokines within the TME represents a promising strategy to maximize local antitumor immune activity while limiting systemic exposure. One approach involves complexing cytokines with the FDA-approved vaccine adjuvant aluminum hydroxide (Alhydrogel^®^ 2% (alum)). Aluminum hydroxide is composed of highly charged aggregates that form a physical depot at the site of injection lasting several weeks (23, 24). Stable cytokine immobilization is achieved by engineering the cytokine payload with an alum-binding peptide (ABP) domain that undergoes phosphorylation on multiple serine residues (25) The phosphorylated ABP then associates with aluminum hydroxide through ligand exchange with surface hydroxyls, and subsequently leads to anchoring of cytokine payloads at the depot site (23, 26–32).

In parallel with preclinical murine studies and human oncology (33), this alum-binding technology has been investigated in dogs with malignant melanoma using JEN-101, a canine IL-12 protein engineered with an ABP domain. JEN-101 was shown to be safe, induced antitumor immunity, and produced objective clinical responses as a single agent when administered intratumorally (34). Treated dogs showed increases in circulating IFN-γ, IL-10, and pro-inflammatory cytokines without systemic adverse events. The observed safety profile of JEN-101 correlated with only insignificant concentrations of canine IL-12-ABP detected in circulation (<1% injected material). At the tumor site, JEN-101 drove strong CD3^+^ T cell recruitment and broad upregulation of inflammatory genes associated with antitumor immunity. JEN-101 is currently being evaluated in additional canine cancers including osteosarcoma, soft tissue sarcoma, mast cell tumor, and mammary gland tumor. Combinatory use of JEN-101 with immune checkpoint blockade deserves exploration in pet dogs with cancer, and is an active field of clinical investigation. The human counterpart of JEN-101, tolododekin alfa (ANK-101), has been evaluated in a first-in-human Phase 1 trial in patients with advanced solid tumors (clinical trial identifier, NCT06171750), demonstrating a similar favorable safety profile, tumor retention of drug, and biological activity including increased intratumoral CD8^+^ T cells and PD-L1 expression (27). Currently, intratumoral tolododekin alfa is being evaluated in combination with cemiplimab for the treatment of cutaneous squamous cell carcinoma.

### 2.2 Collagen-anchored cytokines

Similar to an alum-binding strategy, but instead using naturally occurring extracellular matrix proteins (collagens I, III, and IV) within the TME as anchoring substrates, collagen-binding cytokine strategies have been recently developed and evaluated in both dogs and humans. By engineering collagen-binding peptide sequences to cytokine payloads, the retention of intratumorally injected cytokine fusion proteins within the TME can be achieved (35–41). This collagen-anchoring strategy has been evaluated in pet dogs with soft tissue sarcoma and malignant melanoma. For these intratumoral investigations, caninized IL-2 and IL-12 were fused to a leukocyte-associated immunoglobulin-like receptor (LAIR1) to physically retain the cytokines within the tumor associated extracellular matrix and enhance local antitumor immune activity (21, 42).

In dogs with soft tissue sarcoma, treatment with collagen-anchored IL-2 and IL-12 was well-tolerated, with only Grade 1/2 adverse events reported such as mild fever, thrombocytopenia, and neutropenia. Systemically, elevations in serum IFN-γ were followed by a delayed increase in IL-10. Within injected tumors, increased CD3^+^ T cell infiltration was observed by immunohistochemistry and bulk gene expression profiling revealed upregulation of genes associated with cytotoxic T and NK cell activities, including *Gzmb*, *Ncr1*, and *Nkg7*, as well as immune-recruiting chemokines *Cxcl10* and *Ccl8*. Pro-inflammatory gene expression profiles within injected tumors returned to baseline by eight days after a single dose, coinciding with upregulation of counter-regulatory molecules including *IDO1*, *PD-1*, and *CTLA-4*. Intratumoral injection of a second collagen-anchored cytokine dose was sufficient to re-engage effector gene expressions, supporting the adoption of repeat dosing strategies to sustain durable immune activation. As soft tissue sarcomas are surgically managed, long-term antitumor responses and cytoreductive activities could not be assessed in this study (42), motivating subsequent evaluation in canine oral malignant melanoma.

In dogs with oral malignant melanoma, a dose-escalation trial combining a single 9-Gy radiation fraction followed adjuvantly with six cycles of intratumoral collagen-anchored IL-2 and IL-12 every two weeks was well-tolerated at lower dose cohorts, with adverse events consistent with those previously observed in sarcoma-bearing dogs. Intratumoral cytokine injections produced robust local and regional tumor responses, and median survival across all treated cohorts was 256 days, compared to a historical median of 65 days for untreated canine oral malignant melanoma (43). Objective responses were achieved in dogs receiving well-tolerated dosages; underscoring the clinical applicability for intratumoral localized cytokines via collagen retention to provide therapeutic benefit within a safe dosing range (21).

These canine studies provided provocative clinical evidence supporting the subsequent investigation of collagen-anchored IL-12 and IL-2 in human cancer patients. Key findings from the dog studies mirror the biological rationale underlying the human therapeutic CLN-617, a single-chain fusion protein co-delivering IL-2 and IL-12 via LAIR2-mediated collagen binding (39). CLN-617 is currently being evaluated in a Phase 1 first-in-human trial in patients with advanced solid tumors alone and in combination with pembrolizumab (clinical trial identifier, NCT06035744).

### 2.3 Cytokine-based electrogene therapy

Electroporation uses electric pulses to transiently increase cell membrane permeability, facilitating intracellular delivery of plasmid DNA encoding pro-inflammatory cytokines. When applied intratumorally, this strategy drives local cytokine expression directly by cells within the TME while limiting systemic exposure. In veterinary oncology, similarly to the human field (44–46), immunotherapeutic approaches have mainly been electrogene therapy (EGT) with plasmids encoding IL-12 for the treatment of various solid cancers amenable to targeted electroporation including mast cell tumors, melanoma, adenocarcinoma, fibrosarcoma, and osteosarcoma (47).

Specifically in mast cell tumors, intratumorally treated lesions reduced in size within 1 to 2 weeks following the completion EGT as a single therapy alone, or in combination with adjuvant chemotherapy and/or surgical excision. Immune activation elicited by EGT was supported by increased lymphocyte and plasma cell infiltration within treated lesions. In the treatment of canine mast cell tumors, since EGT was performed using human-coding IL-12 plasmids, plasma concentrations of hIL-12 served as a pharmacokinetic target for measuring systemic cytokine release following local intratumoral EGT in pet dogs. Measurement of systemic IFN-γ in treated pet dogs served as a pharmacodynamic biomarker of EGT’s capacity to elicit effector T cell activation. Intratumoral EGT was safe and supported by the absence of systemic adverse events, IL-12-induced mast cell degranulation, or subsequent histamine release (48). Complementing an intratumoral approach, peritumoral IL-12 gene therapy in canine mast cell tumors also revealed antitumor activity with a favorable safety profile (49). Collectively, these studies in canine mast cell tumors demonstrate the potential of EGT with xenogeneic IL-12 (human) approaches; however, some of the cytoreductive immune reactivity observed in these investigations could be associated with the expression of a foreign protein and not necessarily a specific anticancer response. To mitigate this confounding variable, later studies have utilized plasmid DNA encoding for canine interleukin-12 (50), representing a necessary step toward minimizing anti-drug antibody responses and reduced efficacy associated with non-canine IL-12 constructs. Nonetheless, human cytokine constructs retain meaningful activity in dogs, and their immunogenicity, often delayed and dose-dependent, can be managed through dosing schedule and route (51).

In the equid species, the use of EGT with human IL-12 for the treatment of melanoma metastases in grey horses has also been described (52). In total, 7 horses with 12 established metastatic melanoma lesions were treated intratumorally with EGT. Clinically significant responses were observed (1 CR), and although biological activity was supported by IFN-γ gene transcription within biopsied tumor samples, observed cytoreductive activities were generally transient and incomplete for most treated lesions.

### 2.4 Antibody-cytokine fusion proteins

Antibody-cytokine fusion proteins, also known as immunocytokines, are a class of engineered molecules designed to improve therapeutic index by selectively delivering cytokine payloads to a specific epitope expressed by either tumor cells or within the TME; favoring local antitumor immune activation while reducing on-target, off-tumor toxicities. Several fusion constructs have been developed and extensively investigated including IgGs and antibody fragments (i.e., single-variable chain fragments) linked to cytokine payloads. Cytokines including IL-2, IL-12, IL-15, IFN-γ, TNF-α, and others have been investigated in preclinical murine models and human clinical settings (53).

Two approaches incorporating either IL-12 or IL-2 have been recently described in both human and pet dogs with cancer. The first technology is IL-12 linked to an NHS antibody to increase IL-12 therapeutic safety (54) and the second fusion protein is hu14.18-IL-2, which combines IL-2 with a human monoclonal antibody that recognizes GD2, a cell membrane molecule expressed by tumors (55).

In the first example, NHS (NHS76) is a fully humanized IgG1 antibody directed against necrosis-specific antigens derived from DNA/histone complexes and preferentially expressed within necrotic tumor tissues. The fusion protein is comprised of 2 human (or canine) IL-12 heterodimers linked to the C-terminal region of the antibody heavy chain. Early murine work established NHS-IL12 as a more effective antitumor agent than recombinant free IL-12 in multiple syngeneic models. Responses were dose-dependent and correlated with elevated circulating IFN-γ, enhanced MHC class I expression on splenic DCs, and increased proliferation of splenic and tumor-infiltrating NK cells and splenic CD8^+^ T cells (54). The translational potential of NHS-IL12 was subsequently evaluated in a comparative oncology trial in pet dogs with naturally occurring malignant melanoma. Eighteen dogs received subcutaneous NHS-IL12 across four dose levels (0.4–2.4 mg/m²) in a dose-escalation phase, followed by an expansion cohort at the defined tolerable dose of 0.8 mg/m². Adverse events including thrombocytopenia, hepatic enzymes elevation, fever, and vasculitis were dose-related, with grade 4–5 toxicities elicited at higher doses. Notably, serum IFN-γ levels exceeding 100 pg/mL were observed in dogs treated at 1.6 mg/m² or higher and tracked closely with toxicity onset and severity, reinforcing IFN-γ as both a pharmacodynamic biomarker and a toxicity threshold indicator (56). Serum IL-10 also increased post-treatment alongside IFN-γ as a marker of drug exposure, a finding that has also been observed in other IL-12-targeted therapies in dogs (21, 34, 42) Treatment with NHS-IL12 increased intratumoral CD8+ T cell infiltration without significant changes in peripheral T or B cell populations, and clinical activity was observed with partial responses in two dogs and one patient sustaining a response over nine months (56).

Subsequently to the evaluation of NHS-IL12 in pet dogs, these findings helped inform the conductance of phase I and Ib evaluations of NHS-IL12 in human subjects, as a monotherapy or combined with avelumab in patients with solid tumors (clinical trial identifiers, NCT01417546 and NCT02994953) (57, 58). Notably, key toxicities such as hepatic enzymes elevations, along with the post-treatment IFN-γ/IL-10 pharmacodynamic kinetic signatures observed in dogs, were recapitulated in the first-in-human setting, supporting the translational value of canine models and IFN-γ as a potential biomarker of IL-12-related toxicity.

In a second example, intratumoral administration of hu14.18-IL2, a GD2-directed IL-2 immunocytokine, has been evaluated in pet dogs with malignant melanoma. Combinatorial treatment with intratumoral hu14.18-IL2 and radiation therapy was well tolerated and exerted measurable antitumor activity with an objective response rate of 17% (CR (1) and PR (1) among 12 dogs). Histology and NanoString analysis identified increased intratumoral lymphocytic infiltration and treatment-associated shifts in innate and adaptive immune-cell gene signatures (59). Prior to this veterinary investigation, hu14.18-IL2 had been studied in phase I and II human trials in patients with melanoma (60, 61) and neuroblastoma (62).

### 2.5 PEGylated cytokines

A distinct strategy for improving cytokine therapeutics is conjugation to polyethylene glycol (PEG), which extends circulating half-life, reduces immunogenicity, and increases intratumoral accumulation through enhanced permeation retention effects. These favor properties endowed by pegylation were demonstrated for human TNFα in preclinical studies with PEGylated rHuTNF-α showing markedly prolonged retention time and greater antitumor necrotic effects than unmodified TNF-α in murine models (63).

This pegylated cytokine delivery approach has been evaluated in a phase I study of pet dogs with various spontaneous tumors (64). Fifteen dogs received PEG-hTNF-α, at dosages ranging between 20-30 µg/kg, with dose-limiting toxicities observed in two dogs (coagulopathy, severe hypotension, and vascular leak syndrome) at 30 µg/kg. Aside from self-limiting gastrointestinal events, biologically active doses elicited transient fever and leukopenia, increased intratumoral inflammation and necrosis, and a significant increase in tumor blood flow on dynamic contrast-enhanced MRI. Modest and transient responses were seen in dogs with melanoma, head and neck squamous cell carcinoma, and mammary carcinoma, and one dog with hemangiosarcoma achieved a partial response lasting 3 months.

### 2.6 Liposomal-encapsulated cytokines

Liposomal encapsulation can extend cytokine half-life and limit systemic exposure, and has been particularly useful for delivering cytokines locally to lung tissues via aerosolization. Liposome encapsulated cytokine strategies have been investigated in pet dogs by Khanna and colleagues, who first showed that nebulized IL-2 liposomes were biologically active and well tolerated in canine studies (65). A follow-up phase I/II trial enrolled nine dogs with spontaneous pulmonary metastases or primary lung carcinoma (20) and confirmed that inhaled IL-2 liposomes were non-toxic and exerted durable therapeutic activity with two of four dogs with metastatic osteosarcoma achieving complete regression of pulmonary lesions lasting more than 12 and 20 months. Bronchoalveolar lavage revealed greater cellularity and significant increase in cytolytic activity, with notable enrichment of macrophages, eosinophils and lymphocytes at 15 days post-aerosolization therapy, reflecting local immune activation within the lung microenvironment. These canine results informed appropriate doses for the first human trial of inhaled IL-2 liposomes in human patients with locally advanced or metastatic sarcoma, or other refractory solid tumors, without eliciting significant toxicity, opposite to non-targeted IL-2 clinical use (66).

Complementary approaches with preferential biodistribution and expression of cytokine payloads within the lung parenchyma can be achieved through intravenous delivery of liposomal gene therapy. For example, a phase I study in dogs with osteosarcoma lung metastases receiving intravenous liposome-DNA complexes encoding canine IL-2 demonstrated immune activation and resulted in clinical responses in lung tumor nodules in 3 of 20 treated dogs, with improved overall survival compared to historical controls (67).

### 2.7 Oncolytic viruses expressing cytokines

An emerging approach for localized immune payload delivery is the use of oncolytic viruses (OVs) expressing antitumor cytokines. In this targeted strategy, specific tumor-selective OVs deliver cytokine payloads directly to the TME, locally inducing immune responses while avoiding off-target toxicities associated with systemic cytokine distribution.

In dogs, an oncolytic virus approach includes M032, a non-neurovirulent oHSV-1 engineered to express human IL-12. The virus is designed to infect and kill glioma cells and concurrently drive local IL-12 expression within the TME. A phase I, dose-escalation trial studied the adjuvant intracranial catheter infusion of M032 into the post-operative resection cavity of pet dogs that had incomplete surgical resection of spontaneous high-grade gliomas (68). The median overall survival time from M032 treatment was 151 days, higher than historical controls. No significant adverse events or dose-limiting toxicities were observed. M032 also induced intratumoral mRNA transcription signatures of immune modulation in glioma-treated dogs (69), and this oncolytic viral technology has also been investigated in human clinical trials (70) (clinical trial identifiers, NCT02062827, NSC733972, and NCT07076498).

Another targeted-strategy involves SV-IFNβ-NIS, a recombinant VSV expressing interferon-beta, which was first shown to be well tolerated in healthy beagle dogs, with dose-limiting adverse events observed only at supratherapeutic doses (71). Tolerability and preliminary antitumor activity were subsequently confirmed in client-owned dogs with various spontaneous malignancies (72). In a follow up investigation known as the VIGOR study, neoadjuvant intravenous VSV-cIFNβ-NIS was administered in dogs with naturally occurring osteosarcoma. Intravenous treatment with VSV-cIFNβ-NIS was well tolerated, induced TME changes with increased tumor inflammation following treatment, and a higher T cell immune signature was linked to prolonged survival time (73).

### 2.8 Emerging cytokine delivery platforms

Two additional tissue-targeting cytokine platforms have shown early promises in pet dogs. First, intratumoral IL-12 gene delivery using a dendritic cell-targeted lentivirus (ZVex12) or an IL-12-encoding self-replicating RNA has been evaluated in a small number of dogs with soft tissue sarcoma. Immunobiologic activity was supported by increased CD5-NKp46^+^ NK cells and CD5dimCD8^+^NKp46^+^ cells, and modulated CD8^+^ T cell infiltration and activation markers, although no radiographic responses were observed in this small initial cohort (74). Second, intravesical co-formulation of IL-12 with chitosan (CS/IL-12) has advanced from initial murine preclinical models where intravesical CS/IL-12 was well-tolerated and induced only mild, transient local inflammation without significant systemic IL-12 dissemination (75), into an ongoing Phase 1 dose-escalation study in pet dogs with naturally occurring invasive urothelial carcinoma (76). In pet dogs, the canine IL-12 with chitosan (CS/caIL-12) formulation has been generally well-tolerated, with toxicity markers largely remaining within normal ranges and serum cytokine analyses revealing no substantial systemic immune responses. A single dose-limiting toxicity (grade 3 diarrhea) was observed at the highest dose level tested. This represents the first targeted cytokine approach reported in canine bladder cancer.

## 3 Conclusion

Collectively, the studies summarized here demonstrate that targeted cytokine delivery can achieve meaningful local antitumor immune activity in pet dogs with spontaneous tumors while avoiding systemic toxicity, which has limited the safe adoption of cytokine therapies in both veterinary and human oncology. Whether delivered via aluminum hydroxide depots, collagen anchoring, liposomal encapsulation, immunocytokine fusion, electrogene therapy, or oncolytic viruses, these approaches have generated favorable pharmacodynamic and safety data that have directly shaped or preceded human clinical trials. Common threads across studies include intratumoral T cell infiltration, an IFN-γ/IL-10 response signature, and transient immune activation following single dosing. Given the phenomenon of rebound immunosuppression, repeated dosing of targeted cytokines and combination with immune checkpoint blockade would be predicted to improve treatment outcomes, although the development of tachyphylaxis as a consequence of repeated immune activation warrants further investigation. Continued progress will also require better-characterized canine immune cell references, particularly myeloid and T-cell subsets, supported by single-cell reference datasets and validated canine-specific reagents. As new technologies emerge and combinatorial immunotherapeutic strategies attract greater promise for improving treatment outcomes, pet dogs with naturally occurring cancers will continue to serve as a critical bridge between preclinical discovery and clinical application, with meaningful benefit to both species.
